# High Tumor Mitochondrial DNA Content Correlates With an Improved Patient's Outcome in WHO Grade III Meningioma

**DOI:** 10.3389/fonc.2020.542294

**Published:** 2020-09-18

**Authors:** Lingyang Hua, Tareq A. Juratli, Hongda Zhu, Jiaojiao Deng, Daijun Wang, Shuchen Sun, Qing Xie, Hiroaki Wakimoto, Ye Gong

**Affiliations:** ^1^Department of Neurosurgery, Huashan Hospital, Shanghai Medical College, Fudan University, Shanghai, China; ^2^Department of Neurosurgery, Medizinische Fakultät Carl Gustav Carus, Technische Universität Dresden, Dresden, Germany; ^3^Department of Neurosurgery, Harvard Medical School, Massachusetts General Hospital, Boston, MA, United States; ^4^Department of Critical Care Medicine, Huashan Hospital, Shanghai Medical College, Fudan University, Shanghai, China

**Keywords:** meningioma, MtDNA content, grade III meningioma, prognosis, radiation, malignant meningioma

## Abstract

**Background:** Studies have shown mitochondrial genome content (mtDNA content) varies in many malignancies. However, its distribution and prognostic values in high-grade meningioma remain largely unknown. In this retrospective study, we sought to assess a putative correlation between the mtDNA content and clinical characteristics.

**Methods:** Mitochondrial DNA was extracted from 87 World Health Organization grade III meningioma samples using a qPCR method. The distribution of mtDNA content in WHO grade III meningioma and its correlations with clinical variables were assessed. Furthermore, we prognostic values were also determined.

**Results:** Mean mtDNA content was 617.7 (range, 0.8–3000). There was no mtDNA distribution difference based on the histological subtypes (*P* = 0.07). Tumors with preoperative radiation were associated with lower mtDNA content (*P* = 0.041), whereas no correlations with other clinical variables were observed. A high mtDNA content was associated with significantly better PFS (*P* = 0.044) and OS (*P* = 0.019). However, in patients who received postoperative radiotherapy, low mtDNA content was associated with better PFS (*P* = 0.028), while no difference in OS was observed (*P* = 0.272). Low mtDNA content was also associated with better OS and PFS in subgroups of patients with ER negative status (PFS, *P* = 0.002; OS, *P* = 0.002).

**Conclusions:** Consistent with other tumors, high mtDNA content was associated with better outcome in WHO grade III meningioma in our cohort. However, for patients who received post-operative radiation therapy, low mtDNA content was associated with better PFS. These findings suggest that mtDNA content may further be explored as a potential biomarker for high-grade meningioma patients and for those who received postoperative radiation therapy.

## Introduction

Tumors of the meninges constitute approximately a third of all primary intracranial neoplasms (1). Meningiomas are thought to arise from the arachnoid cap cells of the intracranial and spinal regions. According to the newest WHO 2016 Central Nervous System (CNS) tumor grading criterion, meningiomas are classified to three WHO grades and fifteen histological subtypes (2). Although the majority of meningiomas are benign and slowly growing tumors, there exist a subset of tumors manifesting aggressive and malignant biological behaviors, usually accompanied with higher tumor grade. The histological grading is strongly associated with tumor recurrence and clinical outcome, with five-year survival rates ranging only 28–61% from reported studies. Although grade III meningioma constitutes only a small proportion (1–2%) of all meningiomas, the malignancy and the clinical outcome remains dismal, with a median overall survival ranging from 2.6 to 5.8 years, compared with the other two lower grade meningioma (3–8). Grade III meningioma is defined by a mitotic index equal or higher than 20 mitoses per 10 high power fields and is further classified to three different subtypes based on the histological features, namely anaplastic, papillary, and rhabdoid (2). The anaplastic subtype is the most common in grade III meningioma, representing about 80% of all grade III meningiomas, while the papillary and rhabdoid constitute about 10%, respectively (9).

The mainstream treatment for grade III meningioma involves radical surgical resection followed by stereotactic radiotherapy or radiotherapy (10). Unfortunately, a considerable proportion of grade III meningioma patients still suffer from recurrence despite in time radiation therapy applied (11, 12). Great efforts have been made to investigate potential medical treatment and identify the predictors for radiation sensitivity. To the best of our knowledge, very few have been identified and none of them are applied for clinical use.

Although genetic and cellar studies have identified a number of genes, proteins, and molecules implicated in the tumor genesis, progression and malignant transformation of meningioma during the last several decades, most molecules studied are concerned with nuclear DNA alternations (13). The role of mitochondria in meningioma has not been well deciphered. Mitochondria contain their own genome, encode their own transitional machinery and 13 critical proteins for the oxidative phosphorylation system, which plays a vital role in reactive oxygen production, redox signaling, apoptosis, and many other biological processes (14). Each mitochondrion possesses multiple copies of a mitochondria genome. The functional component of mitochondrial DNA in a cell depends mainly on the content of mitochondrial genomes and the integrity of each mtDNA molecule. Broad ranges of mtDNA content in cells have been reported, from a few in embryonic to several thousands in cardiac myocytes (15). Studies have demonstrated that alterations of mtDNA content are involved in the development and progression of cancer, and the mtDNA content is often changed in tumors compared to non-neoplastic tissues (16–24). Although the content of mtDNA varies in a variety of tumors, it has never been reported in meningioma before, let alone in grade III meningioma. There is only one study investigating the mutational status of mtDNA and they concluded that mtDNA instabilities is relate to tumorogenesis of meningioma (25).

In this retrospective study, we investigated mtDNA content in a cohort of grade III meningioma patients and its association with clinical characteristics, treatment status and prognosis.

## Patients and Methods

### Studies and Patients

Samples from eighty-seven meningioma patients (44 males and 43 females) with WHO grade III meningioma who underwent surgical resection between 2003 and 2008 at the Neurosurgical Department of Huashan Hospital, Fudan University, were included in the current analysis (Table 1). Based on their histology, the meningiomas were assigned to one of three WHO groups: 63 anaplastic, 12 papillary, and 12 rhabdoid meningiomas. Among those, 59 (67.8%) were primary tumors and 28 (32.2%) were recurrent. The histopathological results were independently re-evaluated and confirmed by two experienced neuro-pathologists (Dr. Yin Wang and Dr. Hong Chen) according to the WHO 2016 meningioma grading criterion. All participants gave their written informed consent and ethical approval for the study was obtained from the Human Subjects Institutional Review Board at Huashan Hospital, Fudan University (KY-2012-17).

**Table 1:** Association between mtDNA content and clinicalpathological variables of Grade III meningioma patients.

**Characteristics**	**mtDNA low *n* (%)**	**mtDNA high *n* (%)**	***P***
Gender			0.546
Male	20 (46.5%)	24 (54.5%)	
Female	23 (53.5%)	20 (45.5%)	
Age			0.283
≤60	32 (74.4%)	36 (83.7%)	
>60	11 (25.6%)	8 (18.6%)	
Tumor location			0.107
Skull base	13 (30.2%)	20 (45.5%)	
Non-skull base	30 (69.8%)	24 (54.5%)	
KPS score			0.438
≤80	13 (30.2%)	15 (34.1%)	
>80	30 (69.8%)	29 (65.9%)	
Tumor status			0.223
Primary	27 (62.8%)	32 (67.4%)	
Recurrent	16 (37.2%)	12 (32.6%)	
Preoperative radiation			0.041^*^
Yes	8 (18.6%)	2 (4.5%)	
No	35 (81.4%)	42 (95.5%)	
Malignant transformation			0.520
Yes	7 (16.3%)	8 (25.6%)	
No	36 (83.7%)	36 (74.4%)	
Extent of resection			0.241
GTR	35 (81.4%)	32 (72.7%)	
STR	8 (18.6%)	12 (27.3%)	
ER			0.290
Positive	24 (55.8%)	30 (68.2%)	
Negative	19 (44.2%)	14 (31.8%)	
PR			0.290
Positive	5 (11.6%)	8 (18.2%)	
Negative	38 (88.4%)	36 (81.8%)	
Ki-67			0.093
≤5	10 (23.2%)	17 (38.6%)	
>5	33 (76.7%)	27 (61.4%)	

ER, estrogen receptor; PR, progesterone receptor;

* *p < 0.05 considered statistically significant*.

### DNA Extraction

All paraffin-embedded meningioma tissues used for DNA extraction were reviewed by Dr. Yin Wang to ensure that at least 50% of the cells were neoplastic. DNA was extracted using the GenElute™ FFPE DNA Purification Kit (Sigma-Aldrich, Kenilworth, USA) according to the manufacturer's instructions and reported other authors (26). Briefly, the tissues were treated with xylene first to remove paraffin, and then were subjected to digestion and DNA subsequently isolated from the digested tissues. The concentration and purity of the DNA were assessed using a Nanodrop 2000 spectrophotometer (Thermo Scientific, Wilmington, DE, USA) and all samples were diluted to a concentration of 0.2 ng/μL DNA and stored at −80°C before mtDNA content analysis.

### mtDNA Content

mtDNA content was determined using a quantitative real-time PCR method. This assay measures relative mtDNA content by measuring the ratio of mitochondrial copy number to single copy nuclear gene. The mitochondrially encoded NADH dehydrogenase subunit 1 gene (MT-ND1) and nuclear single copy gene β-globin were used in our study. The specific sequences of primers and TaqMan probes used for the amplification were as follows: MT-ND1 forward (MT-ND1-F), 5′-CCCTAAAACCCGCCACATCT-3′; MT-ND1 reverse (MT-ND1-R), 5′-GAGCGATGGTGAGAGCTAAGGT-3′; β-globin forward (β-globin-1), 5′-GTGCACCTGACTCCTGAGGAGA-3′; β-globin reverse (β-globin−2), 5′-CCTTGATACCAACCTGCCCAG-3′. The TaqMan probes were labeled with 5′-FAM (6-carboxyfluorescein, fluorescent reporter) and 3′-TAMRA (6-carboxy-tetramethylrhodamine, fluorescent quencher). qPCR amplification for each sample was performed using TaqMan™ Universal Master Mix II on the Applied Biosystems (Foster City, CA) Quantstudio 6 real-time PCR system. All samples were run in triplicate for both mitochondrial and nuclear genes on a 384 well plate with a 7500 Fast Real-time PCR system (qRT-PCR; PE7500 real-time PCR instrument; Applied Biosystems, Foster City, CA, USA). Proper positive and negative controls, a calibrator DNA and a standard curve, were also included in each run to monitor the performance of PCR reactions. The thermal conditions for both primers were 95°C for 30 s, followed by 35 cycles of 94°C for 30 s, 58°C for 30 s, and 72°C for 50 s with signal acquisition. The results were analyzed with the 7500v2.0.4 software (Applied Biosystems). The ratio of MT-ND1 copy number to β-globin copy number was calculated for each sample from standard curves. Furthermore, the ratio of each sample was normalized to a calibrator DNA to standardize between different runs, and it was defined as relative mtDNA content.

### Immunohistochemistry

Immuno-histochemical staining was carried out by using monoclonal antibodies, including Ki-67, ER and PR (Signal way [SAB], Shanghai, China; 1:200 dilution). Immuno-histological staining of these antigens was evaluated by two experienced neuro-pathologists as descried above. The tumor was considered ER or PR positive if > 10% of the tumor nuclei showed staining; weak positive when 1–9% of the tumor nuclei were stained; and negative for tumors with no nuclei staining (27, 28).

### Statistical Analysis

All statistical computations used in this study were performed using Stata 13.3 for Windows. mtDNA content distribution was tested with the Shapiro-Wilk test. Fisher's exact test was used to analyze associations between the clinical variables and mtDNA content. The association between mtDNA content and Ki-67 labeling index was tested using the logistic regression model. Continuous variables with skewed distribution were compared with Mann-Whitney U test. Survival curves were drawn as Kaplan-Meier survival plots and analyzed with log-rank test. Cox proportional hazards regression model was applied for the univariate, multivariate and stratified prognostic analysis. A *P* value < 0.05 was considered to be statistically significant.

## Results

### mtDNA Content Distribution and Its Association With Clinical Variables

The mtDNA content was measured using a real-time PCR based method in a cohort of 87 WHO grade III meningiomas. The mean mtDNA quantity was 617.7 (range, 0.8–3000) mtDNA per cell. The mean mtDNA quantity was 955.8 (range, 0.8–3000) in anaplastic meningioma, 988.8 (range, 548.5–2336.8) in papillary subtype, and 612.7 (range, 460.2–1815.2) in rhabdoid subtype. No significant difference in the distribution of mtDNA was observed between the three histological subtypes (*p* = 0.07, Kruskal-Wallis test). Therefore, we combined these three histological subtypes together for further statistical analysis. Linear regression model was performed to see whether the mtDNA distribution is associated with tumor proliferation marker (Ki-67 labeling index). No correlation between these two parameters was observed (*P* = 0.056, *r* = 0.031). Since mtDNA content was not normally distributed (*P* < 0.0001, Shapiro-Wilk test), we classified the cohort into two groups based on the median of mtDNA content (median, 782.2), resulting in mtDNA low group (43 tumors) and high group (44 tumors).

**Figure 1:**
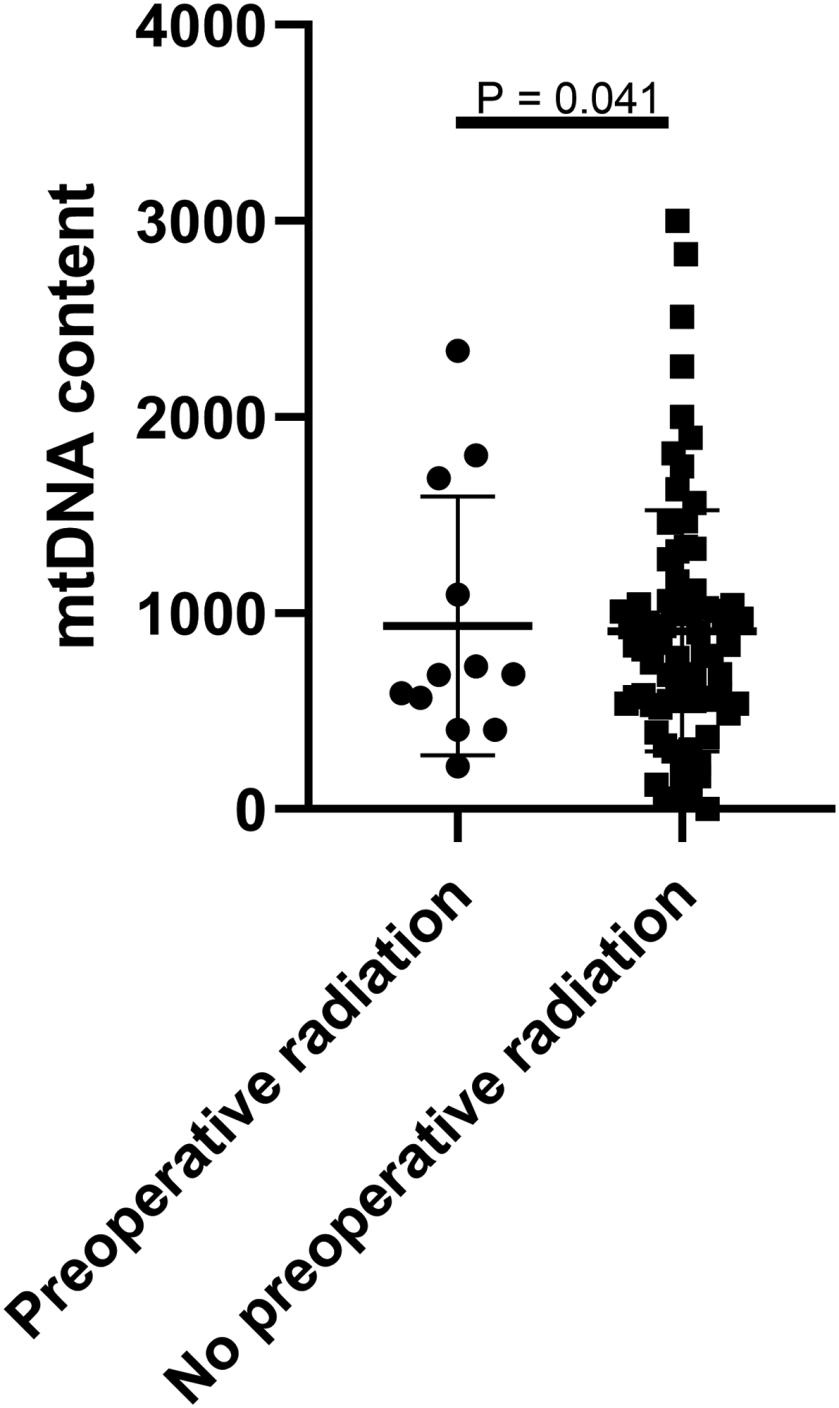
mtDNA content was significantly lower in patients with preoperative radiation therapy.

To evaluate the clinical relevance of mtDNA, we then compared the mean mtDNA with various clinicopathological characteristics. As shown in Table 1, no significant differences were observed between mtDNA and gender, age, tumor location (skullbase vs. convexity), preoperative KPS score, tumor recurrent status, malignant transformation, extent of tumor resection, ER status, PR status, or Ki-67 labeling index (*P* > 0.05, Fisher's exact test). However, there was a significant difference with regard to preoperative radiation therapy; patients who received radiation therapy before surgery (*N* = 10) were more prevalent in the mtDNA low group (Figure 1, *P* = 0.041, Fisher's exact test).

### Prognostic Analysis of mtDNA Content in Grade III Meningioma Patients

**Figure 2:**
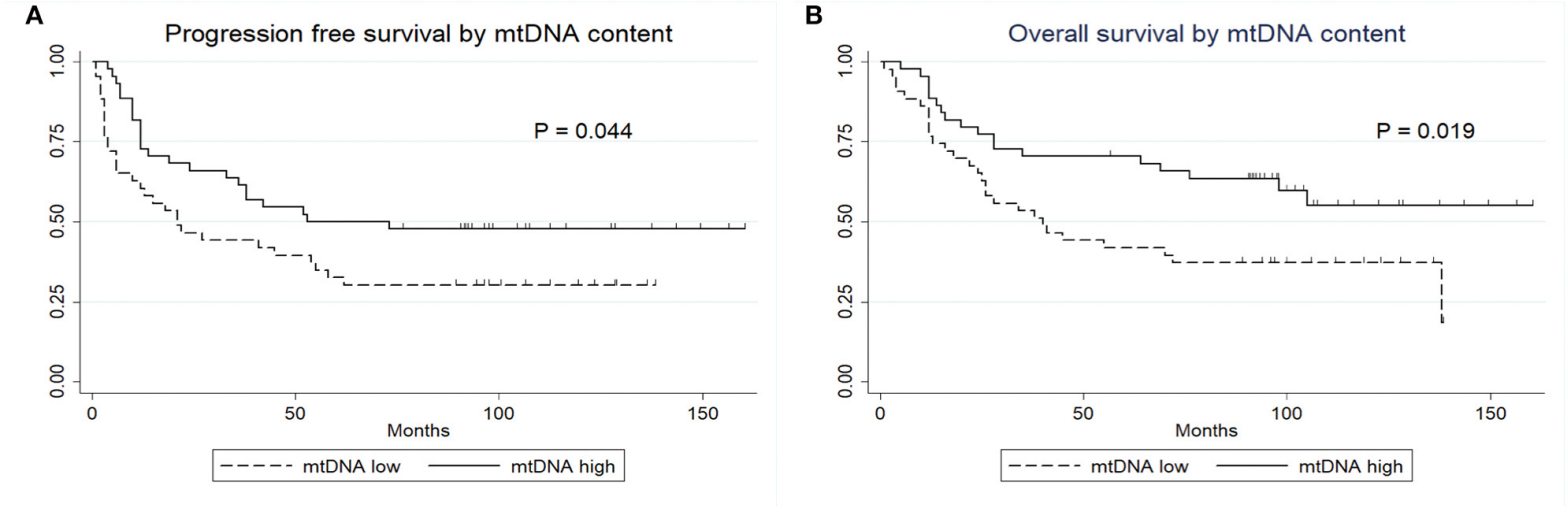
Kaplan-Meier survival curves. **(A)** PFS of patients by mtDNA content. **(B)** OS of patients by mtDNA content.

We next studied the association between mtDNA content and progression free survival (PFS) and overall survival (OS). As shown in Figure 2, patients with high mtDNA content showed a significantly longer PFS (*P* = 0.044, log-rank test) as well as OS (*P* = 0.019, log-rank test). The median PFS in mtDNA low and high group were 43.93 months (range, 1–136 months) and 68.14 months (range, 3–160 months, *p* = 0.049), respectively, while the median OS was 58.19 (range, 1–138 months) and 78.89 (range, 4–160 months, *p* = 0.023), respectively. Univariate Cox proportional hazards model revealed that patients with high mtDNA content had a significantly better outcome regarding extent of resection (GTR) and negative ER (Table 2). We next performed multiple Cox proportional hazards model by testing a number of prognostic factors including ER status (5, 29). Here, we found that along with ER, mtDNA was also an independent prognostic factor for both PFS (*P* = 0.002) and OS (*P* = 0.002) (Table 2).

**Table 2:** Univariate and multivariate analysis of prognostic factors for WHO grade III meningioma patients.

	**Univariate Analysis**				**Multivariate Analysis**			
	**PFS**		**OS**		**PFS**		**OS**	
**Variable**	***p***	**HR (95% CI)**	***p***	**HR (95% CI)**	***P***	**HR (95% CI)**	***p***	**HR(95% CI)**
Age (<60/≥60)	0.819	1.062 (0.636–1.733)	0.974	1.012 (0.502–2.039)				
Gender (female/male)	0.669	0.911 (0.593–1399)	0.275	1.388 (0.252–0.921)				
Preoperative KPS (<80/≥80)	0.877	0.958 (0.555–1.654)	0.987	1.005 (0.540–1.870)				
Extent of resection (GTR/STR)	0.001^*^	0.412 (0.246–0.692)	0.027^*^	0.481 (0.252–0.921)	0.013^*^	2.697 (1.232–5.900)		
Location (skull base/non-skull base)	0.006^*^	1.887 (1.195–2.978)	0.043^*^	1.824 (1.018–3.269)				
Radiation (no/yes)	0.433	0.841 (0.544–1.298)	0.034^*^	0.532 (0.297–0.953)				
Ki-67 index (<5%/≥5%)	0.958	0.988 (0.621–1.570)	0.847	1.064 (0.567–1.995)				
ER (−/+)	0.001^*^	2.884 (1.508–5.514)	0.002^*^	2.966 (1.466–6.001)	0.002^*^	3.366 (1.558–7.271)	0.002^*^	3.523 (1.559–7.962)
PR (−/+)	0.884	0.946 (0.446–2.007)	0.539	0.763 (0.323–1.805)				
Recurrent (no/yes)	0.000^*^	2.532 (1.539–4.167)	0.107	1.637 (0.899–2.981)	0.039^*^	1.841 (1.032–3.283)		
mtDNA content (low/high)	0.049^*^	0.579 (0.336–0.998)	0.023^*^	0.502 (0.277–0.909)	0.002^*^	0.378 (0.202–0.706)	0.002^*^	0.349 (0.178–0.683)

KPS, karnofsky performance score; PFS, progression-free survival; OS, overall survival; HR, hazard ratio; CI, confidence interval; GTR, gross total resection; ER, estrogen receptor; PR, progesterone receptor;

* *p < 0.05 considered statistically significant*.

### Prognostic Analysis of mtDNA Content in Grade III Meningioma Patients Receiving Postoperative Radiation

Radiation therapy has been frequently associated with mitochondria damage (30). Therefore, we analyzed the prognostic factors in patients who received postoperative radiation therapy. A total of 47 patients in our cohort received either gamma knife or external beam radiation after tumor resection. The tumor mtDNA content in patients who underwent postoperative radiation therapy was not normally distributed (*P* < 0.0001, Shapiro-Wilk test). In this subgroup, we further classified patients into mtDNA low and high group based on the median mtDNA content in this group, which was 295.7 per cell. Three patients who previously harbored meningiomas with low mtDNA in the initial 87 patients cohort were reclassified as mtDNA high tumors in this 47 patients group.

**Figure 3:**
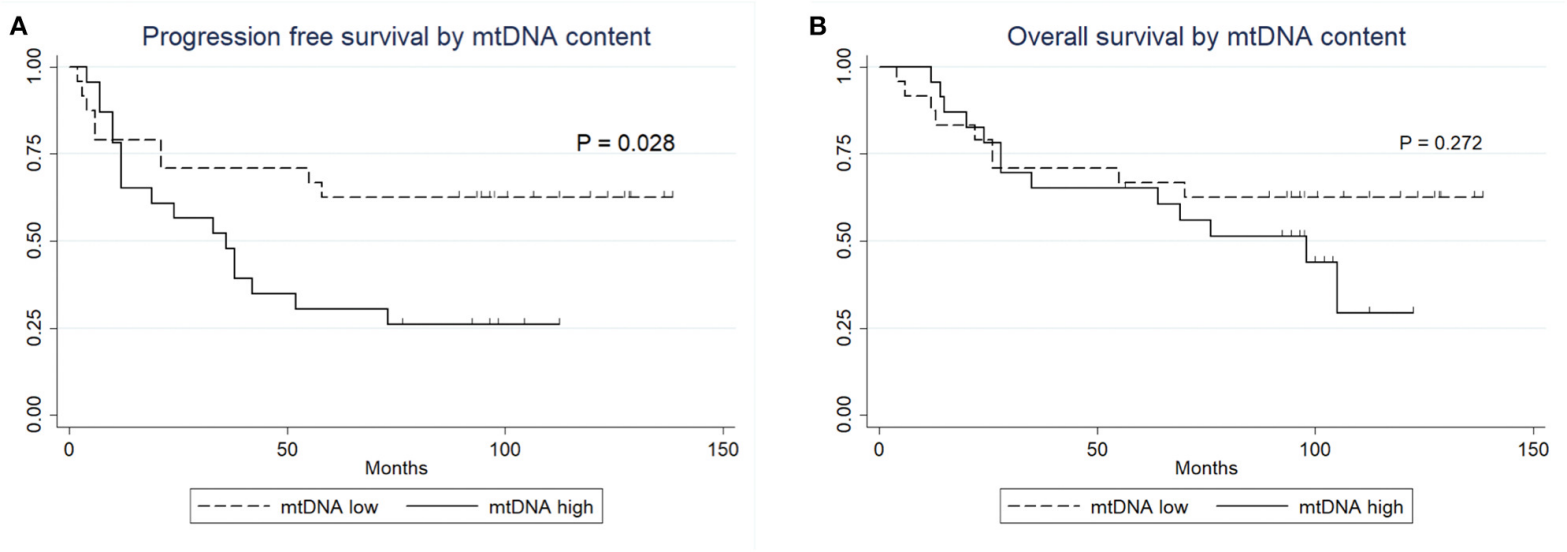
Kaplan-Meier survival curves. **(A)** PFS of patients with post-operative radiation therapy by mtDNA content. **(B)** OS of patients with post-operative radiation therapy by mtDNA content.

**Figure 4:**
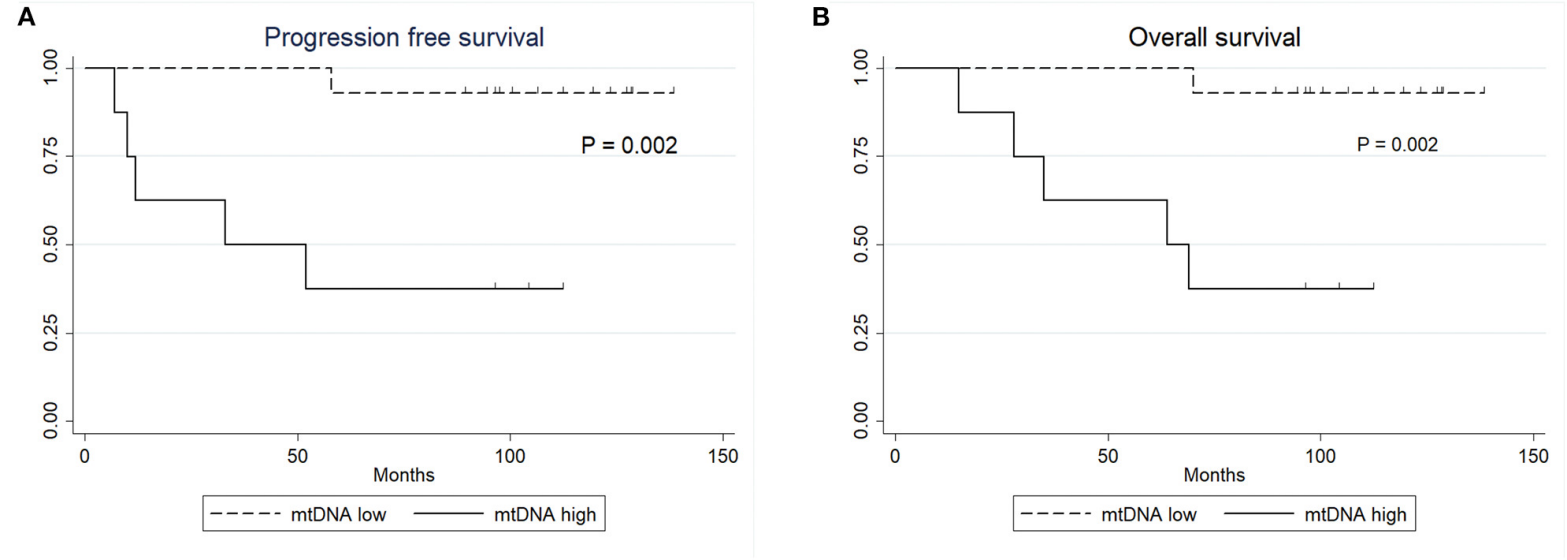
Kaplan-Meier survival curves. **(A)** PFS of patients with ER negative status by mtDNA content. **(B)** OS of patients with ER negative status by mtDNA content.

No statistically significant differences were observed between the clinical variables and the mtDNA content, including preoperative radiation status (*P* > 0.05, Fisher's exact test, Table 3). Interestingly, the univariate Cox proportional survival analysis revealed that low mtDNA content in the irradiated tumor group was associated with better PFS (*P* = 0.035) (Table 4), which was further confirmed by Kaplan-Meier analysis (*P* = 0.028) (Figure 3). However, it was not associated with OS (*P* = 0.272) (Figure 3). Our multivariate analysis of patients with post-operative radiation did not identify mtDNA content as a significant independent factor for PFS or OS, while ER status was found to be a very significant prognostic factor (Table 4). Stratified analysis revealed that, for patients who received postoperative radiation therapy, low mtDNA content was associated with longer OS and PFS in patients with ER negative status (PFS, *P* = 0.002; OS, *P* = 0.002) (Figure 4).

**Table 3:** Association between mtDNA content and clinical-pathological variables of Grade III meningioma patients who receive post-operative radiation therapy.

**Characteristics**	**mtDNA low *n* (%)**	**mtDNA high *n (*%)**	***P***
Gender			0.096
Male	16 (66.7%)	13 (56.5%)	
Female	8 (33.3%)	10 (43.5%)	
Age			0.609
≤60	19 (79.2%)	18 (78.3%)	
>60	5 (20.8%)	5 (21.7%)	
Tumor location			0.092
Skull base	6 (25.0%)	11 (47.8%)	
Non-skull base	18 (75.0%)	12 (52.2%)	
KPS score			0.598
≤80	6 (25.0%)	6 (26.1%)	
>80	18 (75.0%)	17 (73.9%)	
Tumor status			0.340
Primary	18 (75.0%)	15 (65.2%)	
Recurrent	6 (25.0%)	8 (34.8%)	
Preoperative radiation			0.525
Yes	4 (16.7%)	3 (13.0%)	
No	20 (83.3%)	20 (87.0%)	
Malignant transformation			0.375
Yes	5 (20.8%)	3 (13.0%)	
No	19 (79.1%)	20 (87.0%)	
Extent of resection			0.221
GTR	20 (83.3%)	16 (69.6%)	
STR	4 (16.7%)	7 (30.4%)	
ER			0.092
Positive	10 (41.7%)	15 (65.2%)	
Negative	14 (58.3%)	8 (34.8%)	
PR			0.325
Positive	3 (12.5%)	5 (21.7%)	
Negative	21 (87.5%)	18 (78.3%)	
Ki-67			0.234
≤5	6 (25.0%)	9 (39.1%)	
>5	18 (75.0%)	14 (60.9%)	

*ER, estrogen receptor; PR, progesterone receptor*.

**Table 4:** Univariate and Multivariate Analysis of Prognostic Factors for WHO grade III meningioma patients receiving post-operative radiation therapy.

	**Univariate Analysis**				**Multivariate Analysis**			
	**PFS**		**OS**		**PFS**		**OS**	
**Variable**	***P***	**HR (95% CI)**	***p***	**HR (95% CI)**	***p***	**HR (95% CI)**	***p***	**HR (95% CI)**
Age (<60/≥60)	0.984	0.991 (0.397–2.471)	0.625	0.763 (0.258–2.258)				
Gender (female/male)	0.528	0.778 (0.357–1.696)	0.774	0.884 (0.382–2.048)	0.013^*^	0.272 (0.097–0.762)		
Preoperative KPS (<80/≥80)	0.854	0.922 (0.387–2.194)	0.960	0.976 (0.381–2.501)				
Extent of resection (GTR/STR)	0.009^*^	2.970 (1.313–6.720)	0.250	1.738 (0.678–4.456)				
Location (skull base/non-skull base)	0.025^*^	2.421 (1.117–5.244)	0.084	2.106 (0.904–4.906)				
Ki-67 index (<5%/≥5%)	0.354	0.688 (0.312–1.518)	0.515	0.749 (0.313–1.790)				
ER (−/+)	0.001^*^	4.875 (1.937–12.270)	0.008^*^	3.587 (1.393–9.238)	0.000^*^	10.202 (3.019–34.468)	0.002^*^	6.692 (2.056-21.784)
PR (−/+)	0.218	1.779 (0.711–4.453)	0.831	1.126 (0.380–3.334)				
Recurrent (no/yes)	0.006^*^	2.994 (1.371–6.539)	0.049^*^	2.362 (1.005–5.553)				
mtDNA content (low/high)	0.035^*^	2.414 (1.066–5.466)	0.278	1.607 (0.682–3.783)				

KPS, karnofsky performance score; PFS, progression-free survival; OS, overall survival; HR, hazard ratio; CI, confidence interval; GTR, gross total resection;

* *p < 0.05 considered statistically significant*.

## Discussions

WHO grade III meningioma are rare and represent 1–2% of all meningiomas. Initial standard-of-care therapy for malignant meningiomas is surgical resection followed by radiation therapy. Nevertheless, the majority patients with malignant meningiomas devlope recurrent tumors and have a dismal outcome (3, 5). Extensive efforts have been made to explore possible prognostic factors that can predict the outcome in malignant meningiomas (31–36). In addition, we previously reported that ER is an independent negative prognostic factor for WHO grade III meningioma (5). In this study, we explored the distribution of mtDNA content and its prognostic value in a total of 87 patients with WHO grade III meningioma. We demonstrated that patients with high intratumoral mtDNA content had a significantly better outcome regarding both OS and PFS. However, in the subgroup of patients who received postoperative radiation therapy, low mtDNA content was associated with better PFS, although it was not an independent prognostic factor in multivariate analysis.

mtDNA content variation has been reported to play an important role in the development of several malignancies. As early as 1996, Liang et al. demonstrated high mtDNA content is associated with more proliferative abilities (37). Since then, an increasing number of studies focused on mtDNA copy number variation and its correlations with tumors. In our cohort, mtDNA content distribution was not associated with the Ki-67 labeling index. This is in concordance with previous studies that demonstrated no correlation between the Ki-67 labeling index and outcomes in grade III meningioma (5, 38). In tumors, the mtDNA content has been shown to be altered compared to adjacent non-neoplastic tissues, including breast, esophageal and prostate cancers (16–18). In addition, a progressive decrease in mtDNA content has been observed during malignant transformation in glioma, breast, prostate, endometrial and head and neck cancers (17–19, 21, 22, 39). We analyzed distribution of mtDNA content between the three histological subtypes within the WHO grade III meningioma and no difference was observed, which was consistent with our previous study demonstrating no difference in the clinical features between the three distinct histological subtypes (5). We did not separately analyze the mtDNA content by the histological subtype was that there were two few papillary and habdoid meningioma patients in the cohort which would lead to statistical bias. Zhang et al. revealed that in their series of 151 glioma patients, mtDNA content was associated with recurrent status in glioma, which was confirmed by two recent studies (40, 41). In breast cancer, Marjolein reported low mtDNA content was more prevalent in ER positive breast cancers (20). In our cohort, however, we did not find any correlations between mtDNA content and major tumor clinical characteristics including tumor recurrence, malignant transformation, and ER status. Nevertheless, low mtDNA content was more prevalent in patients who received preoperative radiation therapy, which may be explained by the radiation-induced DNA damage in the mitochondrial genome combined by impaired enzyme synthesis.

Although most studies revealed that high mtDNA content was associated with better outcome in cancer, some studies showed contrary data (18, 42–51). Thus, the role of mtDNA in cancer prognosis remains controversial. Consistent with the results reported in breast, prostate, and esophageal cancer and glioma, we found a strong correlation between outcome and mtDNA content in our cohort. High mtDNA content was associated with both better PFS and OS of patients with grade III meningioma. In addition, along with ER status, mtDNA content was an independent prognostic factor for both PFS and OS, suggesting that alterations in mitochondria genome might be associated with progression in high grade meningioma.

Interestingly, our survival analysis demonstrated that in patients who received postoperative radiation (*n* = 47), low tumor mtDNA content was associated with better PFS, whereas no OS difference was observed. Consistently, studies in breast cancer demonstrated that low tumor mtDNA content is associated with better outcome after receiving chemotherapy due to induction of mitochondrial damage. Likewise, as radiation can induce mitochondrial damage (52), a suitable hypothesis is that tumors with low mtDNA content may be more susceptible to mitochondrial damage induced by radiation than those with high mtDNA content.

A recent work by Soon et al. reported that mitochondrial DNA is mutated in several glioma cells and could subsequently induce mitochondrial dysfunction and higher oxidative stress (53). Mitochondrion dysfunction has been theorized as a crucial player in many tumors by increasing the production of reactive oxygen species (ROS) through the activity of the mitochondrial electron transport chain (54). This altered cellular respiratory system may generate excessive reactive oxygen species, and further lead to a vicious cycle of DNA damages. This hypothesis was also proved in pediatric high-grade glioma in a recent study by Shen et al. (41). In their study, higher mtDNA content was associated with better outcome while reducing mtDNA content induced inhibited tumor growth. Reducing mtDNA content in combination with radiation demonstrated synergistic effect. Their study gave us a hint that in grade III meningioma, treatment strategies using pharmacological inhibition of mtDNA content in combination of radiation could also be effective. Pharmacological agents such as DCA can reduce tumor mtDNA content and inhibit meningioma growth, while reduced mtDNA content may render these tumors more sensitive to postoperative radiotherapy.

The main limitation of our study is the small patient number included, especially in the radiation subgroup, which may limit the statistical power of survival and stratified analyses. Moreover, meningioma patients with lower histological grade were excluded. Consequently, the distribution of mtDNA content in different grades of meningioma cannot be evlautaed.

## Conclusions

In conclusion, our study highlights the prognostic association of mtDNA content in WHO grade III meningioma. High mtDNA content was an independent prognostic factor for better PFS and OS. However, for patients who received postoperative radiation therapy, low mtDNA content was associated with better PFS. Tumor mtDNA content may serve as a marker to predict the outcome of patients with WHO grade III meningioma. Further studies are warranted to explore the mtDNA distribution in lower WHO grade tumors and the possible role of the mitochondrial genome in the progression of meningioma and response to radiation therapy.

## Data Availability Statement

The raw data supporting the conclusions of this manuscript will be made available by the authors, without undue reservation, to any qualified researcher.

## Ethics Statement

The studies involving human participants were reviewed and approved by Huashan Hospital Fudan University. The patients/participants provided their written informed consent to participate in this study.

## Author Contributions

LH, HZ, YG, and QX: study concepts. LH: study design and manuscript preparation, quality control of data and algorithms. LH, HZ, JD, and SS: data acquisition. LH, TJ, HW, and QX: data analysis and interpretation. LH, TJ, and HW: statistical analysis. TJ, HW, and QX: manuscript editing. HW, YG, and QX: manuscript review. All authors have read and agreed to the published version of the manuscript.

## Conflict of Interest

The authors declare that the research was conducted in the absence of any commercial or financial relationships that could be construed as a potential conflict of interest.

## Abbreviations

mtDNA: mitochondrial genome content
GTR: gross total resection
STR: subtotal resection
WHO: World Health Organization
ER: estrogen receptor
PR: progesterone receptor
PFS: progression free survival
OS: overall survival.
